# FAIM: An Antagonist of Fas-Killing and Beyond

**DOI:** 10.3390/cells8060541

**Published:** 2019-06-04

**Authors:** Jianxin Huo, Shengli Xu, Kong-Peng Lam

**Affiliations:** 1 Bioprocessing Technology Institute, Agency for Science, Technology and Research, Singapore 138668, Singapore; huo_jianxin@bti.a-star.edu.sg (J.H.); xu_shengli@bti.a-star.edu.sg (S.X.); 2 Department of Physiology, Yong Loo Lin School of Medicine, National University of Singapore, Singapore 117593, Singapore; 3 Department of Microbiology and Immunology, Yong Loo Lin School of Medicine, National University of Singapore, Singapore 117545, Singapore; 4 School of Biological Sciences, Nanyang Technological University, 60 Nanyang Drive, Singapore 637551, Singapore

**Keywords:** FAIM, B cells, Fas-mediated apoptosis, TCR-mediated apoptosis, metabolism, Alzheimer’s disease, Multiple myeloma, Akt, c-FLIP

## Abstract

Fas Apoptosis Inhibitory Molecule (FAIM) is an anti-apoptotic protein that is up-regulated in B cell receptor (BCR)-activated B cells and confers upon them resistance to Fas-mediated cell death. *Faim* has two alternatively spliced isoforms, with the short isoform ubiquitously expressed in various tissues and the long isoform mainly found in the nervous tissues. FAIM is evolutionarily conserved but does not share any significant primary sequence homology with any known protein. The function of FAIM has been extensively studied in the past 20 years, with its primary role being ascribed to be anti-apoptotic. In addition, several other functions of FAIM were also discovered in different physiological and pathological conditions, such as cell growth, metabolism, Alzheimer’s disease and tumorigenesis. However, the detailed molecular mechanisms underlying FAIM’s role in these conditions remain unknown. In this review, we summarize comprehensively the functions of FAIM in these different contexts and discuss its potential as a diagnostic, prognostic or therapeutic target.

## 1. Introduction

Apoptosis is a form of programmed cell death that plays important roles in various physiological processes to maintain homeostasis by controlling the number of cells in the tissues. It is an irreversible process that can be initiated through either an intrinsic or extrinsic pathway. In the intrinsic pathway, the cell “kills” itself upon sensing cell stress, whereas in the extrinsic pathway, the cell “kills” itself after receiving death signals from other cells. Both pathways trigger a cascade of enzymatic activation of proteins called caspases (cysteine-aspartic proteases) that destroy the cell by degrading proteins indiscriminately. Fas (CD95/APO-1/TNFRSF6), a member of the tumor necrosis factor (TNF) receptor superfamily, is a death receptor that mediates the extrinsic apoptosis of cells [1]. Engagement of Fas receptors by FasL (CD178) leads to Fas trimerization and the formation of the intracellular death-inducing signalling complex (DISC) that comprises adapter protein Fas-associated death domain protein (FADD) and procaspase-8 (FADD-like interleukin-1β-converting enzyme (FLICE)). Pro-caspase-8 is activated by auto-cleavage within DISC and subsequently leads to the activation of the downstream caspase-3 followed by the execution of apoptosis. In the immune system, Fas plays important roles in regulating lymphocyte maturation, receptor repertoire selection and the deletion of autoreactive lymphocytes to ensure a functional and innocuous immune system in humans. Fas-mediated apoptosis is tightly regulated by both positive and negative signals to ensure proper Fas killing of lymphocytes for the regulation of lymphocyte growth and differentiation [2,3]. Fas apoptosis inhibitory molecule (FAIM) has been identified as a negative regulator of Fas signalling [4] and was subsequently found to play multifaceted roles in many physiological processes. In this review, we will discuss the physiological role of FAIM and its involvement in pathological situations such as tumorigenesis and metabolic disorders.

## 2. The Discovery of FAIM

FAIM was first identified as a negative regulator of Fas signalling in BCR-stimulated B lymphocyte. Using mRNA differential display, Schneider et al. found that *faim* gene was inducibly expressed in murine primary B lymphocytes stimulated with antibodies directed against their surface immunoglobulin M (IgM) and in the presence of soluble dimeric CD40 ligand (CD40L). Consequently, FAIM is upregulated and enables the B cells to be resistant to Fas-mediated apoptosis. It was also found that FAIM is a relatively small molecule of 179 amino acids in length and is ubiquitously expressed [4].

Subsequent study revealed that *faim* gene, which consists of six exons and is located at chromosome 9f1 in mice (syntenic region 3q22.3 in humans), gives rise to two alternatively spliced RNA isoforms that share part of the 5′UTR in exon I but have two different translation initiation sites in exons II and exon III, respectively. The longer splicing variant of *faim* (FAIM-L) contains 22 additional amino acids on the *N*-terminus compared to the smaller variant (FAIM-S) [5]. FAIM-S is found to be ubiquitously expressed in all tissues, whereas FAIM-L is specifically expressed in neural tissues, such as the cortex, cerebellum, hippocampus, hindbrain and spinal cord [6].

**Figure 1:**
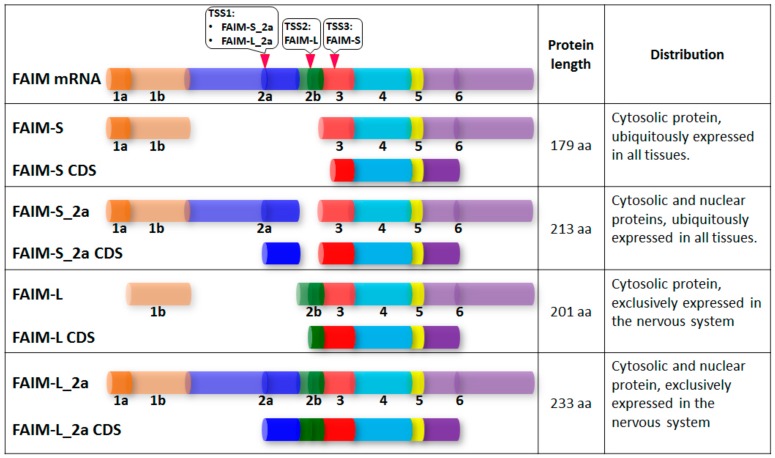
Structure and comparison of four human Fas apoptosis inhibitory molecule (FAIM) isoforms. Schematic representation of FAIM gene structure shown with exon 1a, 1b, 2a, 2b, 3, 4, 5 and 6. In contrast to the 5′ and 3′ untranslated regions (UTRs), coding sequence (CDS) regions are indicated with a darker color. TSS, Transcriptional start site.

More recently, Coccia et al. found two additional FAIM isoforms, FAIM-S_2a and FAIM-L_2a, which have the same sequence as FAIM-S and FAIM-L but include exon 2a. Similar to the expression patterns of FAIM-S and FAIM-L, FAIM-S_2a is ubiquitously expressed in all tissues, whereas FAIM-L_2a is exclusively expressed in the nervous system. Interestingly, in contrast to cytosolic proteins FAIM-S and FAIM-L, FAIM-S_2a and FAIM-L_2a are able to localize to the nucleus, indicating that these two FAIM isoforms may have additional neural-related functions (Figure 1). Importantly, FAIM-S_2a and FAIM-L_2a increased neurite outgrowth in NGF-treated PC12 cells, indicating their functional role in neuronal development and differentiation [7].

## 3. Structural Study of FAIM

Although FAIM is highly conserved evolutionarily, it does not share significant primary sequence homology with any known protein. NMR spectroscopy study of murine FAIM-S revealed that its N- and C-regions can fold independently of each other and clearly constitute distinct domains [8]. Detailed analysis revealed that the C-terminal domain of FAIM shares some topological features with the “up-and-down barrel” motif found in fatty acid binding protein (FABP) and the P2 family of proteins. However, unlike FABP, the C-terminal domain of FAIM appears to be too small to accommodate a fatty acid residue within its cavity and probably has no role in lipid binding. The architecture of the C-terminal domain (CTD) of FAIM is also similar to that of type III fibronectin domains except for the lack of β-strands found in the latter. The lack of structural homology to other proteins suggests that the FAIM-CTD structure cannot directly shed light on the molecular mechanisms of FAIM [8]. Another study of the structure of human FAIM-S showed that the crystal structure of the N-terminal domain (NTD) of FAIM and the NMR solution structure of the C-terminal domain adopt a similar protein fold containing eight antiparallel β-strands which form two sheets. Further structural and biochemical analyses implied that the N-terminal domain exists as a dimer and the C-terminal domain exist as a monomer and that they can interact with each other. A mutation study suggested that residues Glu38, Arg110 and Asn123 are important for the interaction of the two domains and the anti-apoptotic activity of FAIM, suggesting that the inter-domain interaction of FAIM is important for FAIM’s anti-apoptotic function [8].

## 4. Physiological Functions of FAIM

### 4.1. FAIM’s Role in Fas-Mediated Apoptosis of B Cells, Thymocytes and Hepatocytes

**Figure 2:**
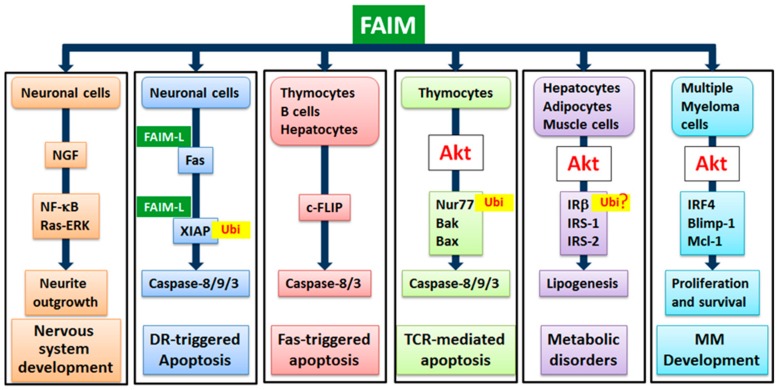
Schematic representation of FAIM’s involvement in various signaling pathways and physiological or disease outcomes.

FAIM was initially found to be up-regulated in BCR-stimulated B cells and antagonized Fas-mediated apoptosis of B cells. Overexpression of FAIM in the B lymphoma cell line BAL-17 confers these cells with resistance to Fas-killing [4]. In contrast, FAIM-deficient B cells exhibited increased sensitivity to Fas-triggered apoptosis in vitro [9]. In addition, FAIM-deficient thymocytes and hepatocytes were also more susceptible to Fas-killing, and FAIM-deficient mice suffered greater mortality and exhibited exacerbated liver damage in response to Fas engagement in vivo. In Fas-mediated apoptosis, trimerized Fas receptor recruits the adapter molecule FADD and caspase-8 into the DISC, resulting in caspase-8 activation. A molecule with sequence homology to caspase-8, termed cellular FLICE-inhibitory protein (c-FLIP), possesses apoptosis-inhibiting functions by binding to DISC to block caspase-8 activation and thereby inhibits Fas-mediated apoptosis [3,10]. Detailed biochemical analyses further revealed decreased expression of c-FLIP (L) (long) and c-FLIP (R) (short) in FAIM-deficient thymocytes and hepatocytes and increased association of caspase-8 with Fas in the mutant cells. These results suggest that FAIM modulates Fas-mediated apoptosis through influencing the expression of c-FLIP and regulating the physical association of caspase-8 and DISC [9] (Figure 2).

### 4.2. FAIM’s Role in TCR-Mediated Apoptosis of Thymocytes

Apoptosis also plays an important role in lymphocyte development and homeostasis [11]. T cells develop in the thymus and undergo a process of positive and negative selection triggered by the engagement of their T cell receptors (TCR) to select for functional and non-auto reactive T cells [12,13]. Further studies demonstrated that FAIM is also up-regulated in thymocytes upon TCR engagement. FAIM-deficient thymocytes were highly susceptible to TCR-mediated apoptosis with increased activation of caspase-8 and -9 in vitro compared with wild-type T cells. In vivo injection of anti-CD3 antibodies mimicking negative selection led to the augmented depletion of CD4^+^CD8^+^ T cells in the thymus of FAIM-deficient compared with wild-type mice, suggesting that FAIM plays an important role in thymocyte apoptosis [14]. Interestingly, engagement of the TCR on FAIM-deficient thymocytes caused elevated protein expression levels of proapoptotic Bcl-2 homologous antagonist/killer (Bak), Bcl-2-associated X protein (Bax) and the orphan steroid nuclear receptor Nur77 (also known as NGFIB or NR4A1) that plays an important role in thymocyte apoptosis [15,16]. In the absence of FAIM, the ubiquitination and degradation of the Nur77 protein was reduced, which was ascribed to defective TCR-induced activation of Akt of which its activity is important for Nur77 ubiquitination. By utilizing FAIM-deficient primary thymocytes and FAIM-overexpressing DO-11.10 T cells, it was demonstrated that FAIM acts upstream of Akt in TCR signalling and facilitates Akt’s localization to lipid rafts, hence affecting its activation [14] (Figure 2).

### 4.3. FAIM’s Dual Functions in the Nervous System

FAIMs’ roles, including those of FAIM-L and FAIM-S, in the nervous system were intensively studied by Comella and colleagues [17]. Their elegant studies demonstrated that FAIM could exert either anti-apoptotic or pro-growth function mediated by different FAIM isoforms and under different contexts.

FAIM-L was shown to protect neuronal cells from Fas-induced apoptosis [18]. Expression of FAIM-L could be induced by nerve growth factor (NGF) through the extracellular signal-regulated kinase (ERK) pathway in neuronal PC12 cells. Neuronal cells overexpressing FAIM-L were resistant to apoptosis induced by death receptors such as Fas or TNFR1, whereas knockdown of endogenous FAIM-L by specific short hairpin RNA (shRNA) caused the neurons to be susceptible to receptor-induced cell death. Mechanistically, FAIM-L-mediated antagonism of apoptosis was ascribed to its direct association with Fas which could prevent the activation of the initiator caspase-8 triggered by Fas [18]. More recently, Moubarak et al. demonstrated that FAIM-L protects rat neuronal Type II cells from Fas-induced apoptosis through regulating X-linked inhibitor of apoptosis (XIAP) which is the most potent caspase inhibitor in vitro and plays an important role in preventing Fas-mediated apoptosis [19,20,21]. In this study, it was shown that FAIM-L could sustain endogenous levels of XIAP in murine cortical neurons. By interacting with the baculovirus IAP repeat (BIR)2 domain of XIAP, FAIM-L could inhibit XIAP auto-ubiquitination and maintain its stability, thus conferring protection from apoptosis [22]. Additionally, by stabilizing XIAP protein levels, FAIM-L could modulate synaptic transmission and prevent chemical induced long-term depression (LTD) induction in mouse hippocampal neurons [23] (Figure 2).

On the other hand, although FAIM-S-overexpression did not rescue the apoptosis of neurons induced by trophic factor deprivation, it could promote neurite outgrowth by activating Ras-ERK (extracellular signal-regulated kinase) and NF-κB (nuclear factor kappa-light-chain-enhancer of activated B cells) pathways [6]. It was shown that FAIM-S overexpression greatly enhanced neurite outgrowth of PC12 cells and sympathetic neurons cultured with NGF, whereas knockdown of FAIM by siRNA decreased neurite outgrowth in these cells. It was further demonstrated that FAIM-S overexpression promoted NF-κB activation and FAIM-S-induced neurite outgrowth was abolished by blockade of NF-κB activation either by using a super-repressor of IκBα or by deleting the p65 NF-κB subunit. It was also demonstrated that the effect of FAIM on neurite outgrowth was blocked by inhibition of the Ras-ERK pathway and FAIM can associate with both TrkA and p75 neurotrophin receptor NGF receptors in a ligand-dependent manner [6]. These studies uncover a novel function of FAIM in promoting neurite outgrowth by a mechanism involving the activation of the Ras-ERK and NF-κB pathways (Figure 2).

### 4.4. FAIM’s Involvement in Myocardial Infarction

Clinical intramyocardial implantation of mesenchymal stem cells (MSCs) can induce neovascularisation and improve heart function after myocardial infarction [24,25]. However, the survival and engraftment of MSCs following transplantation remain as challenges to this therapeutic approach. Recent research showed that Silent information regulator 2 homolog 1 (SIRT1) could rejuvenate and improve the therapeutic efficacy of aged rat MSCs. Interestingly, it was also found that the protective effect of SIRT1 activator SRT1720 on aged human MSCs was mediated by FAIM [26]. FAIM was significantly upregulated by SRT1720 treatment and the knockdown of FAIM by siRNA can block the anti-apoptotic effect of SRT1720, suggesting that FAIM is involved in improving the survival of aged hMSCs and enhancing the therapeutic efficacy of myocardial infarction mediated by SRT1720 [26].

### 4.5. FAIM’s Role in Insulin Signalling and Energy Homeostasis

Recently, FAIM was also demonstrated to play a non-redundant role in insulin signaling that is known to trigger multiple signaling cascades to regulate various cellular activities, such as glucose metabolism, cell proliferation and energy metabolism [27]. Upon binding by insulin, the intrinsic tyrosine kinase activity of the insulin receptor (IR) is stimulated and this leads to IR auto-phosphorylation followed by the phosphorylation of the insulin receptor substrates (IRS)-1 and IRS-2 proteins and the downstream adaptor protein Src-homology/collagen (SHC). These events result in the phosphorylation of phosphatidylinositol 3-kinase (PI3-kinase) and growth factor receptor-bound protein 2 (Grb2) to activate PI3-kinase and MAPK signaling pathways [28]. Importantly, IR beta (IRβ) ubiquitination followed by its proteasome degradation is critical for insulin signaling [29]. In the absence of FAIM, IRβ was markedly reduced in all insulin-sensitive tissues such as hepatocytes, adipocytes and skeleton muscle cells. Consequently, the phosphorylation of IRS-1 and Akt2 were largely attenuated in these tissues in response to insulin treatment. At the same time, lipogenesis was elevated in the liver of FAIM-deficient mice as indicated by increased fatty acid synthesis and sterol regulatory element-binding protein-1 (SREBP-1) and SREBP-2 activation [27]. These results suggest that FAIM is a novel regulator of insulin signaling, probably by regulating the protein turnover of IRβ, and plays an important role in energy homeostasis (Figure 2).

## 5. The Involvement of FAIM in Diseases

### 5.1. Multiple Myeloma

Multiple myeloma (MM) is an incurable hematological malignancy characterized by the accumulation of tumorigenic plasma cells in the bone marrow where they receive signals from the tumor microenvironment for survival and proliferation [30]. Interferon regulatory factor 4 (IRF4) was identified as a myeloma-associated proto-oncogene transcriptional factor. IRF4 is frequently involved in t (6;14)(p25;q32) chromosomal translocation in multiple myeloma [31,32,33]. Interestingly, FAIM’s expression was found to be up-regulated in IRF4-overexpressing IL-3-dependent murine B cell line Ba/F3 [34]. Later, it was revealed that IRF4 directly binds to the promoter of *faim* and upregulates the expression of *faim*. Furthermore, FAIM also enhances CD40-induced IRF4 expression in B cells, suggesting that the induction of FAIM initiates a positive reinforcing feedback loop in which IRF4 expression is both enhanced by and further promotes FAIM expression.

By examining a panel of MM cell lines, Huo et al found that FAIM is expressed in MM cells, albeit at different levels [35]. More importantly, the survival of MM cells was significantly reduced upon the knocking down of FAIM expression by siRNA. Interesting, knockdown of FAIM also led to decreased levels of B lymphocyte-induced maturation protein (Blimp-1) and Myeloid cell factor-1 (Mcl-1), which are critical for multiple myeloma survival [36,37]. These studies suggest that FAIM plays a critical role in MM cell survival by modulating the expression of critical survival proteins and transcription factors.

Consistent with this view, Huo et al. demonstrated that FAIM expression is upregulated in MM cells upon their treatment with insulin-like growth factor-1 (IGF-1) [35]. IGF-1 is a major myeloma growth factor and regulates mitogenesis and glucose metabolism and potentiates plasma cell malignancy [38]. It was found that FAIM could also mediate IGF-1-induced IRF4 upregulation as knockdown of FAIM resulted in decreased IRF4 induction by IGF-1 treatment. Interestingly, knockdown of FAIM compromised Akt activation which was required for IGF-1-induced IRF4 upregulation. Taken together, these results suggest that an IRF4-FAIM-Akt-IRF4 signaling axis functions downstream of IGF-1 signaling in MM cells [35].

The clinical relevance of FAIM was studied in normal and MM samples using primary gene expression data obtained from a patient cohort (15 healthy individuals and 147 patients with various plasma cell neoplasm including 22 cases of monoclonal gammopathy of undetermined significance (MGUS), 24 of smoldering MM (SMM) and 101 of symptomatic MM) [39,40]. It was found that FAIM expression was increased in MM patients as compared with normal individuals. More importantly, FAIM expression was significantly higher in symptomatic MM patients compared with asymptomatic and premalignant individuals. The impact of FAIM expression levels on patient survival was examined in a large clinical cohort. In one cohort of 351 newly diagnosed myeloma patients treated with high-dose chemotherapy and stem cell transplantation, high FAIM expression was correlated with significantly shorter survival. In another cohort of patients who had relapsed disease and were treated with the proteasome inhibitor Velcade or Bortezomib, higher FAIM expression was also correlated with poorer overall survival [35]. These studies demonstrate the diagnostic and prognostic relevance of FAIM expression in MM.

### 5.2. Myeloproliferative Diseases

Chronic myeloproliferative disorders (MPDs) are clonal haematopoietic stem cell malignancies with accumulation of mature myeloid cells in the bone marrow and peripheral blood. Various studies have demonstrated that the deregulation of apoptosis related genes and their regulatory molecules play critical roles in the pathogenesis of MPDs [41,42]. A recent study examining gene expression levels of a panel of death receptors and related apoptotic genes including *faim* in CD34 hematopoietic stem cells and leukocytes from patients with MPDs [43] found that the expression of *faim* was elevated together with that of *fas*, *fasl* and *c-flip*, suggesting that the deregulation of *faim* expression might associate with MPD pathogenesis and the accumulation of myeloid cells in MPDs.

### 5.3. Other Solid Tumors

Recent studies also indicate that FAIM could be involved in solid tumors. For instance, a previous gene-profiling study showed that FAIM’s expression was significantly downregulated in human pancreatic cancer cells upon their treatment with histone deacetylase inhibitor that could induce the apoptosis of these cells [44]. In a study of the importance of microRNAs (miRNA) in prostate cancer, the expression of miR-133b was found to be significantly downregulated in 75% of the cases when compared with matched healthy tissues [45]. Interestingly, FAIM was revealed as an immediate target of mir-133 in prostate cancer cells, indicating a potentially important role for FAIM during cellular transformation and tumorigenesis in prostate cancer. FAIM was also shown to be a potential epigenetic modifier in esophageal cancers. Ahrens et al. found that FAIM was significantly downregulated in esophageal cancer cells after combinatory treatment of histone deacetylase and DNA methyltransferase inhibitors [46]. The explicit role of FAIM in cancer development probably depends on specific cell type and tissues and still remains not completely understood. Therefore, more overarching research is required to better delineate the specific role of FAIM in each type of cancer.

### 5.4. Obesity and Hepatosteatosis

Recently, FAIM was demonstrated to be involved in metabolic disorders such as obesity and hepatosteatosis. Even on normal chew diet, mice deficient in FAIM spontaneously developed nonhyperphagic obesity [27]. The mutant mice also manifested hepatosteatosis, adipocyte hypertrophy, dyslipidaemia, hyperglycaemia and hyperinsulinaemia. Studies of FAIM-deficient mice further demonstrated that enhanced lipogenesis is probably the cause of these metabolic disorders. In particular, saturated fatty acid [C16:0] was significantly increased in the adult liver tissue of mutant mice. In addition, monounsaturated fatty acids ([C16:1], [C18:1], [C20:1], [C22:1] and [C24:1]), polyunsaturated fatty acids ([C20:2], [C18:3], [C20:3], [C18:4] and [C22:4]) were all markedly elevated in the mutant mice. Detailed study revealed that FAIM-deficiency led to enhanced expression of sterol regulatory element binding protein (SREBP)-1a and SREBP-1c and their downstream lipogenic target genes such as stearoyl-CoA desaturase 1 (SCD-1), fatty acid synthase (FAS) and acetyl-CoA carboxylase (ACC). Concordantly with the higher level of cholesterol in blood, the SREBP-2 pathway, which preferentially activates cholesterol synthesis in the liver by targeting 3-hydroxy-3-methyl-glutaryl-CoA reductase (HMGCR), was also elevated in FAIM-deficient hepatocytes.

The role of FAIM in metabolism was also explored in obese patients. Studies of a small clinical cohort that consisted of 33 obese patients and 14 lean volunteers demonstrated that FAIM expression was lower in obese patients and FAIM expression level was reversely correlated with insulin resistance biomarkers. In future, more comprehensive studies of larger cohorts of patients with obesity and hepatosteatosis are necessary for a full understanding of how FAIM regulate lipid metabolism and energy homoeostasis.

### 5.5. Alzheimer’s Disease

FAIM has also been implicated in neuronal conditions. Carriba et al. reported that FAIM-L was reduced in hippocampal samples from patients with Alzheimer’s disease (AD). The expression of genes involved in neuron degeneration such *caspase-8, c-flip, lifeguard and faim-L* was examined systematically using postmortem hippocampal samples from AD patients. Among the genes analyzed, *faim-L* was the most clearly altered during the progression of Braak stages which belong to the widely used postmortem analysis to describe the amount and distribution of neurofibrillary tangles correlated with the severity of dementia [47]. To further elucidate FAIM’s role in AD, they examined a mouse model of AD and found that FAIM expression was reduced in the entorhinal and hippocampal cortex before the onset of neurodegeneration. In mouse primary cortical neurons, amyloid-β (Aβ) reduced FAIM-L expression, suggesting that FAIM reduction is associated with the progression of AD. Mechanistically, TNFα protection against Aβ toxicity was suppressed by knockdown of *faim-L* by RNA interference (RNAi) or by Aβ treatment. In fact, the quantity of FAIM-L was shown to affect the response of neurons to inflammatory stimulus such as TNFα cytokine. These studies suggest that FAIM plays a protective role in AD [48,49]. Given the involvement of FAIM in AD where neuronal cell survival plays a critical role, it is not far-fetched to imagine that FAIM could also be involved in Parkinson’s disease (PD).

### 5.6. Intellectual Disability

Intellectual disability (ID) is a genetically heterogeneous neurodevelopmental disorder. Large-scale sequencing has led to the identification of more than 700 candidate genes involved in ID, reflecting the complexity of the molecular mechanism involved [50]. It has been hypothesised that *de novo* variation in the three prime untranslated region (3′-UTR) might contribute to ID pathogenesis [51]. Studies have discovered 44 single nucleotide non-coding variants within the 3′UTR of 50 severe ID patient genomes with high-confidence, with 3′UTR of *faim* being one of the non-coding variants. Interestingly, *faim*’s 3′UTR was predicted to harbour binding sites for mir-140-3p, and the expression of this 3′UTR was significantly down-regulated by mir-140-3p. This study suggests that *de novo* FAIM 3′UTR variant and its functional consequences might be a primary cause for ID (Table 1).

## 6. Conclusions and Future Perspectives

Since the identification of FAIM by Schneider et al. in 1999, significant progress has been achieved in understanding the physiological role of FAIM. The involvement of FAIM in different physiological and disease contexts is summarized in Table 1. It is not difficult to imagine that FAIM has multifaceted roles under different conditions given its ubiquitous and evolutionally conserved expression. In fact, its involvement in metabolic dysfunction and tumorigenesis suggests that FAIM could be used as an important diagnostic or prognosis marker. FAIM has potential as a target for the development of novel therapies for the treatment of these critical diseases. However, so far, there is no FAIM-interacting protein identified, which makes it difficult to determine its structure and precisely understand its molecular mechanisms for its function. Therefore, in the future, there is an urgent need to identify FAIM’s interacting partners in different physiological and pathological contexts.

## Figures and Tables

**Table 1:** Summary of FAIM’s involvement in diseases.

Conditions	Tissue/Cell	FAIM Expression and/or Effects	FAIM’s Roles and Mechanisms	Ref.
Multiple myeloma (MM)	IRF4-expressing multiple myeloma cell lines	FAIM was upregulated in IRF4-expressing MM cells.	IRF4-FAIM plays roles in MM progression.	[34]
	MM patients and MM cell lines	FAIM expression correlates with poorer survival outcomes of newly diagnosed MM patients treated with stem cell transplantation or relapsed MM patients treated in clinical trials with Bortezomib.	FAIM’s diagnostic and prognostic value in MM patients.	[35]
		FAIM is required in MM cells for their survival. FAIM is induced by IGF-1 or IL-6. FAIM mediates IGF-1 induced Akt activation. IRF4 upregulation by IGF-1 is mitigated by FAIM-KD or inhibition of Akt.	FAIM-IRF4-Akt forward feedback loop for MM development.	
Myeloproliferative diseases (MPD)	CD34 cells and leukocytes from MPD patients	FAIM is elevated in CD34 cells obtained from MPD patients.	FAIM may contribute to MPD pathogenesis.	[43]
Prostate cancer	Prostate cancer patients and PC3 cell line	FAIM is one of miR-133b immediate targets.	FAIM may contribute to prostate tumorigenesis and tissue homeostasis.	[45]
Esophageal cancers	Esophageal squamous cell carcinoma (ESCC) and esophageal adenocarcinoma (EAC) cells	Inhibition of histone deacetylases downregulates FAIM expression.	FAIM is one of various genes regulated by inhibition of histone deacetylases in esophageal cancer cells.	[46]
Obesity and hepatosteatosis	Human and mouse	FAIM defects lead to non-hyperphagic obesity accompanied by hepatosteatosis, adipocyte hypertrophy, dyslipidaemia, hyperglycaemia and hyperinsulinaemia SREBP-1a and SREBP-1c upregulation SCD-1, FAS, ACC and HMGCR upregulation	FAIM mediates insulin signaling and plays an essential role in energy homoeostasis. IRβ, IRS-1 and IRS-2 stability	[27]
Alzheimer’s disease (AD)	Human and mouse	FAIM-L was reduced in hippocampal samples from AD patients. FAIM-L was altered during the progression of BRAAK stages of AD. TNFα protection against Aβ toxicity was suppressed when the FAIM-L expression level was reduced by RNA interference (RNAi).	FAIM is associated with the progression of AD.	[48]
Intellectual disability	Intellectual disability patients	FAIM is down-regulated in intellectually disabled patients.	Unknown	[51]

## Abbreviations

Aβ, amyloid-β; AD, Alzheimer’s disease; ACC, acetyl-CoA carboxylase; Bak, proapoptotic Bcl-2 homologous antagonist/killer; Bax, Bcl-2-associated X protein; BCR, B cell receptor; BIR, baculovirus IAP repeat; Blimp-1, B lymphocyte-induced maturation protein; Caspase, cysteine-aspartic protease; CD40L, CD40 ligand; CDS, coding sequence; c-FLIP, cellular FLICE-inhibitory protein; CTD, C-terminal domain; DISC, death-inducing signalling complex; ERK, extracellular signal–regulated kinase; FABP, fatty acid binding protein; FADD, Fas-associated death domain protein; FAIM, Fas Apoptosis Inhibitory Molecule; FAS, fatty acid synthase; FLICE, FADD-like interleukin-1β-converting enzyme; Grb2, growth factor receptor-bound protein 2; HMGCR, 3-hydroxy-3-methyl-glutaryl-CoA reductase; IGF-1, insulin-like growth factor-1; ID, intellectual disability; IgM, immunoglobulin; IR, insulin receptor; IRF4, interferon regulatory factor 4; IRS, insulin receptor substrates; Mcl-1, myeloid cell factor-1; MGUS, monoclonal gammopathy of undetermined significance; miR, microRNAs; MM, Multiple myeloma; MPDs, myeloproliferative disorders; MSCs, mesenchymal stem cells; NF-κB, nuclear factor kappa-light-chain-enhancer of activated B cells; NGF, nerve growth factor; NGFIB, nerve growth factor IB; NPC, normal plasma cells; NR4A1, nuclear receptor subfamily 4 group A member 1; NTD, N-terminal domain; Nur77, also known as NGFIB or NR4A1; P2 family, P2 myelin protein; PD, Parkinson’s disease; PI3-kinase, phosphatidylinositol 3-kinase; RNAi, RNA interference; SCD-1, stearoyl-CoA desaturase 1; SHC, Src-homology/collagen; shRNAi, short hairpin RNAi; SIRT1, silent information regulator 2 homolog 1; SMM, smoldering MM; TCR, T cell receptors; TNF, tumor necrosis factor; TSS, Transcriptional start site; UTRs, untranslated regions; XIAP, X-linked inhibitor of apoptosis
